# Muscle MRI in neutral lipid storage disease (NLSD)

**DOI:** 10.1007/s00415-017-8498-8

**Published:** 2017-05-13

**Authors:** Matteo Garibaldi, Giorgio Tasca, Jordi Diaz-Manera, Pierfancesco Ottaviani, Francesco Laschena, Donatella Pantoli, Simonetta Gerevini, Chiara Fiorillo, Lorenzo Maggi, Elisabetta Tasca, Adele D’Amico, Olimpia Musumeci, Antonio Toscano, Claudio Bruno, Roberto Massa, Corrado Angelini, Enrico Bertini, Giovanni Antonini, Elena Maria Pennisi

**Affiliations:** 1 Unit of Neuromuscular Diseases, Department of Neurology Mental Health and Sensory Organs (NESMOS), Faculty of Medicine and Psychology, ‘Sapienza’ University of Rome, Sant’Andrea Hospital, Rome, Italy; 2 Institute of Neurology, Policlinico “A.Gemelli” Foundation University Hospital, Rome, Italy; 3 Neuromuscular Diseases Unit, Department of Neurology, Hospital de la Santa Creu i Sant Pau, Universitat Autònoma de Barcelona (UAB), Barcelona, Spain; 4 Centro de Investigación Biomédica en Red de Enfermedades Raras (CIBERER), Barcelona, Spain; 5 Department of Radiology, Istituto Dermopatico dell’Immacolata, IRCCS, Rome, Italy; 6 Neuroradiology Unit, Department of Radiology, San Filippo Neri Hospital, Rome, Italy; 7 Neuroradiology Department, IRCCS San Raffaele Hospital, Milan, Italy; 8 Pediatric Neurology and Muscular Disorders, Istituto Giannina Gaslini, Genoa, Italy; 9 Neuroimmunology and Neuromuscular Diseases Unit, Fondazione IRCCS Istituto Neurologico Carlo Besta, Milan, Italy; 10 IRCCS S.Camillo, Lido di Venezia, Italy; 11 Unit of Neuromuscular and Neurodegenerative Disorders, Laboratory of Molecular Medicine, ‘Bambino Gesù’ Children’s Hospital, IRCCS, Rome, Italy; 12 Department of Clinical and Experimental Medicine, University of Messina, Messina, Italy; 13 Department of Systems Medicine (Neurology), University of Rome Tor Vergata, Rome, Italy; 14 Department of Neurology, San Filippo Neri Hospital, Rome, Italy

**Keywords:** NLSD, PNPLA2, ABHD5, Lipid storage disease, Muscle MRI

## Abstract

Neutral lipid storage disease (NLSD) is a rare inherited disorder of lipid metabolism resulting in lipid droplets accumulation in different tissues. Skeletal muscle could be affected in both two different form of disease: NLSD with myopathy (NLSD-M) and NLSD with ichthyosis (NLSD-I). We present the muscle imaging data of 12 patients from the Italian Network for NLSD: ten patients presenting NLSD-M and two patients with NLSD-I. In NLSD-M gluteus minimus, semimembranosus, soleus and gastrocnemius medialis in the lower limbs and infraspinatus in the upper limbs were the most affected muscles. Gracilis, sartorius, subscapularis, pectoralis, triceps brachii and sternocleidomastoid were spared. Muscle involvement was not homogenous and characteristic “patchy” replacement was observed in at least one muscle in all the patients. Half of the patients showed one or more STIR positive muscles. In both NLSD-I cases muscle involvement was not observed by T1-TSE sequences, but one of them showed positive STIR images in more than one muscle in the leg. Our data provides evidence that muscle imaging can identify characteristic alterations in NLSD-M, characterized by a specific pattern of muscle involvement with “patchy” areas of fatty replacement. Larger cohorts are needed to assess if a distinct pattern of muscle involvement exists also for NLSD-I.

**Electronic supplementary material:**

The online version of this article (doi:10.1007/s00415-017-8498-8) contains supplementary material, which is available to authorized users.

## Introduction

Neutral lipid storage disease (NLSD) is a rare inherited disorder of lipid metabolism characterized by a defect in the catabolic pathway of triacylglycerols resulting in systemic accumulation of triglycerides in cytoplasmic droplets, notably in the leukocytes (*Jordan’s anomaly*). Two different recessive forms have been described: (1) the NLSD with myopathy (NLSD-M) caused by molecular defects in the adipose triglyceride lipase gene (*ATGL*, also called patatin-like phospholipase domain-containing 2, *PNPLA2*) coding for a rate-limiting enzyme catalyzing the first step of hydrolysis of triglycerides [1], and (2) the NLSD with ichthyosis (NLSD-I or *Chanarin*-*Dorfman Disease*) due to mutations in the *ABHD5* gene (also known as Comparative Gene Identification-58, *CGI*-*58*) coding an homonymous activator protein of ATGL [2].

Typical NLSD-I presentation include an early onset ichthyosis (nonbullous congenital ichthyosiform erythroderma, NCIE) associated with liver and mild skeletal muscle involvement [3]. Clinically NLSD-M is mainly characterized by adult-onset progressive myopathy with variable association of cardiomyopathy, hepatic steatosis and short stature. Muscle weakness is diffuse, but frequently predominant in proximal upper limb and distal lower limb muscles, leading to a “man in the barrel” phenotype frequently associated with neck extensor weakness [4].

In the last years, evidence is accumulating on the usefulness of muscle imaging in defining specific patterns of muscle involvement in inherited muscle diseases to help clinicians in the diagnostic workup [5–7]. Globally, metabolic myopathies have not yet been widely investigated and most of the studies concern Pompe Disease, which present a characteristic pattern of muscle involvement [8–10].

Muscle imaging in NLSD have not been systematically investigated. Only few studies reported muscle MRI findings in small series of patients with NLSD-M [4, 11, 12]. They showed a heterogeneous involvement mainly affecting the posterior compartment of the thighs, anterior and posterior compartment of the legs, deltoid, trapezius, infra- and supraspinatus. By contrast, typical NLSD-I shows milder involvement although cases presenting with severe and diffuse fatty replacement of muscles have been described [13].

The aim of this study was to assess the skeletal muscle involvement by muscle imaging (MRI or/and CT) in a cohort of NLSD patients from the Italian network for NLSD, and in particular to establish whether there is a consistent pattern of muscle involvement.

## Materials and methods

### Patients

Seven Italian Neuromuscular Centers participating to the Italian Network of NLSD were involved in patients’ enrolling. Patients having at least a complete muscle imaging study (CT scan or MRI with both T1 and STIR sequences) of lower limbs were included. Patients without complete muscle imaging study were asked to undergo to muscle MRI or CT scan in their referring neuromuscular center according to the agreed imaging protocol. Finally, a total of 12 patients (10 NLSD-M and 2 NLSD-I) from 9 different families met the inclusion criteria for enrolment. Clinical severity at time of imaging was assessed by neurologists considering the overall muscle involvement.

### Muscle imaging

Muscle scans were obtained by MRI (10 patients) or computed tomography (CT) studies (2 patients) in five different Neuromuscular Centers for most patients in accordance to international consensus recommendation [14, 15]. MRI images were obtained using 1.5-T MR scanners and both T1-TSE and STIR sequences were analyzed. Complete study of lower limbs was available for all patients. Axial slices of scapular girdle were available from seven patients. Coronal and sagittal slices of upper and lower limbs were analyzed in available studies as well. The proximal part of the upper limbs was analyzed when evaluable. Standard whole-body CT scans (spacing 10–45 mm; total mAs 12,074, total DLP 2200 mGy cm) were performed when MRI was contraindicated previous informed consent.

A total of 33 muscles of lower limbs were analyzed from each side in all patients and 18 muscles of scapular girdle in 7 patients (listed in Supplementary Materials). Both cervical and thoracic paraspinal muscles were evaluated as one muscle for each segment as well as the anterior arm muscles (biceps brachii, coracobrachialis and brachialis) because they could not be reliably distinguished in all patients. Each muscle was evaluated throughout its length either with MRI or CT studies using a five point scale (0–4) according to Fischer classification [16]. Scans were independently evaluated by two experienced neurologists (MG and EMP) blind to clinical data. Muscle involvement was considered asymmetric when the score difference between the two sides was at least of 2 points [17].

Scans were also analyzed evaluating the overall pattern of involvement and comparing the results to previously described studies [4, 11, 12].

## Results

### Patients

Twelve patients from nine families were enrolled in the study. All but one patient (patient 6 with Iranian origin) had Italian origin. All patients (10 NLSD-M and 2 NLSD-I) had genetically confirmed recessive mutations (5 homozygotes and 5 compound heterozygotes for PNPLA2 mutation, and 2 homozygotes for ABDH5 mutations). Complete clinical data of patients has been previously reported [3, 18–24]. Briefly, NLSD-M patients showed a mild-to-severe muscle weakness and both NLSD-I patients had no skeletal muscle manifestations. Histological and biochemical findings were typical for all cases. The main clinical and molecular data are summarized in Table 1. Clinical severity at time of imaging was assessed considering the overall muscle involvement.

**Table 1:** Clinical data of NLSD patients

Patient	Sex	Clinical form	Age at onset (muscle involvement) (years)	Age at imaging (years)	Phenotype (muscle weakness)	Severity (at time of imaging)	References
1	M	NLSD-M	40	62	Proximal LL	Mild, ambulant	[13]
2	M	NLSD-M	35	50	Distal UL, LL	Moderate, ambulant	[14]
3	M	NLSD-M	34	50	Diffuse UL, LL, axial	Severe, ambulant with support	[15]
4	M	NLSD-M	35	45	Diffuse UL, LL, axial	Severe, ambulant with support	[15]
5	M	NLSD-M	40	44	Distal LL	Mild, ambulant	[16]
6	F	NLSD-M	18	52	Diffuse UL, LL, axial	Severe, wheelchair-bound	[15]
7	M	NLSD-M	40	62	Proximal UL, LL, axial	Moderate, ambulant	[14]
8	M	NLSD-M	5	15	Asymptomatic	Very mild, ambulant	[17]
9	M	NLSD-M	1	25	Proximal UL, LL	Moderate, ambulant	[18]
10	F	NLSD-M	58	74	Proximal LL	Mild, ambulant	[19]
11	M	NLSD-I	–	15	Asymptomatic	Very mild, ambulant	[3]
12	F	NLSD-I	–	26	Asymptomatic	Very mild, ambulant	[3]

*UL* upper limbs, *LL* lower limbs, *very mild* not symptomatic patients, *mild* ambulant patients with weakness in max 2 districts (distal or proximal in upper or lower limbs), *moderate* ambulant patients with weakness in more than 2 districts, *severe* ambulant with support or wheelchair patients with diffuse muscle weakness

### Lower limb muscles

**Fig. 1:**
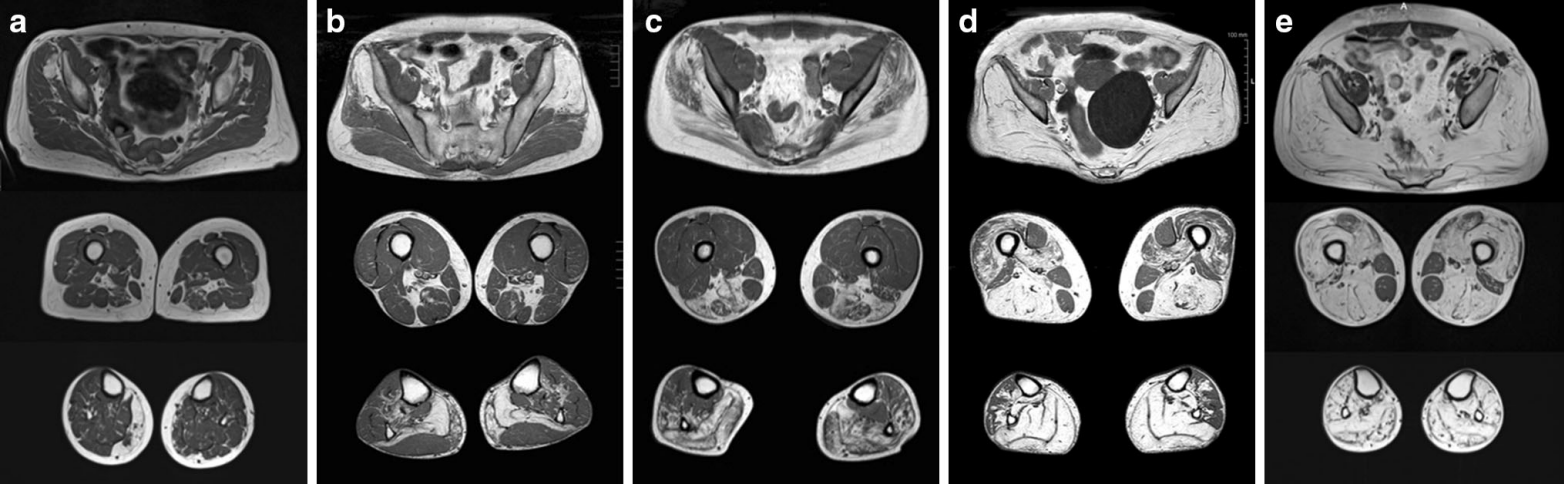
Lower limbs involvement in different stages of NLSD-M. T1-weighted images of lower limbs in NLSD-M patients of different severities: **a** P10, 74 years; **b** P1, 62 years; **c** P9, 25 years; **d** P6, 52 years; **e** P4, 45 years. Gluteus minimus, semimembranosus, gastrocnemius medialis and soleus are constantly affected muscles even if in different extents. Leg muscles are invariably more affected than thigh. Posterior compartment of both leg and thigh show more severe involvement than anterior compartment. Psoas, biceps femoris (short head) and rectus femoris show a mild involvement in late-disease course. Gracilis and sartorius are constantly spared

All NLSD-M patients showed fatty replacement in the following 4 muscles, which were affected in all stages of disease: gluteus minimus, semimembranosus, soleus, and gastrocnemius medialis. This common pattern was often associated with the involvement of the gluteus medius, biceps femoris (long head), tibialis posterior and tibialis anterior (8/10 patients). Gastrocnemius lateralis was affected in 6/10 patients, but in all subjects fatty replacement was moderate or severe (score 3–4). Lumbar paraspinal muscles were also frequently affected (8/10 patients) (Fig. 1).

**Fig. 2:**
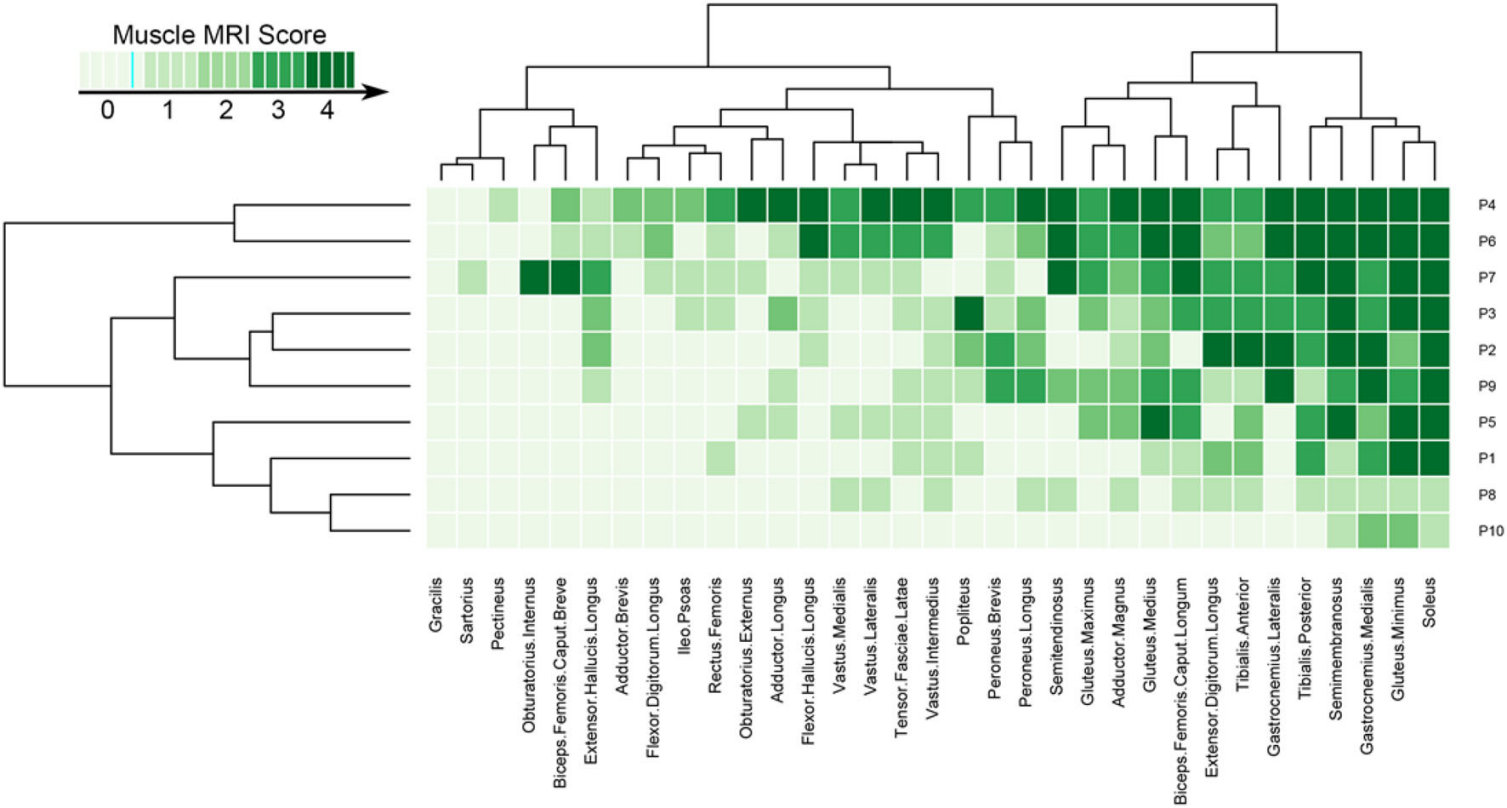
Heatmap of lower limbs involvement in NLSD-M

Conversely, gracilis, sartorius and pectineus were spared even in later stages of disease. Biceps femoris (short head), adductor brevis, and iliopsoas were also frequently spared (7/10 patients). Quadriceps was always relatively spared compared with muscles of the posterior thigh compartment. The overall muscle involvement of lower limbs in NLSD-M patients is schematized heatmap table (Fig. 2).

Half of the patients showed at least two STIR positive muscles.

In both NLSD-I cases fatty replacement was not detected by T1-TSE sequences, but one patient showed positive STIR images in the legs (Fig. 4c).

### Scapular girdle muscles

**Fig. 3:**
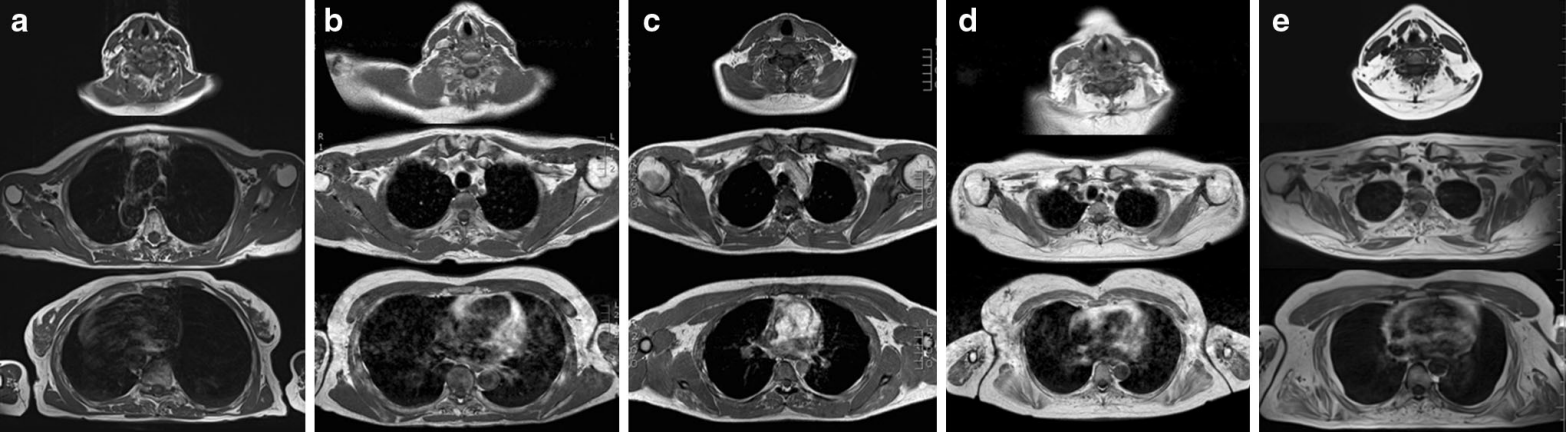
Scapular girdle involvement in NLSD-M. T1-weighted images of lower limbs in NLSD-M patients of different severities: **a** P10, 74 years; **b** P1, 62 years; **c** P9, 25 years; **d** P6, 52 years; **e** P4, 45 years. Infraspinatus, trapezius, deltoid and thoracic paraspinous were the most affected muscles and subscapularis, pectoralis minor and major, and sternocleidomastoid were the most spared. Pectoralis major and sternocleidomastoid sometimes had a hypertrophic appearance (**e**). Note that least affected patients (P10, **a**; P1, **b**) showed no T1-hyperintense muscle in scapular girdle. Interestingly infraspinatus showed STIR positive signal in P1 (**b**) (see Fig. 4)

**Fig. 4:**
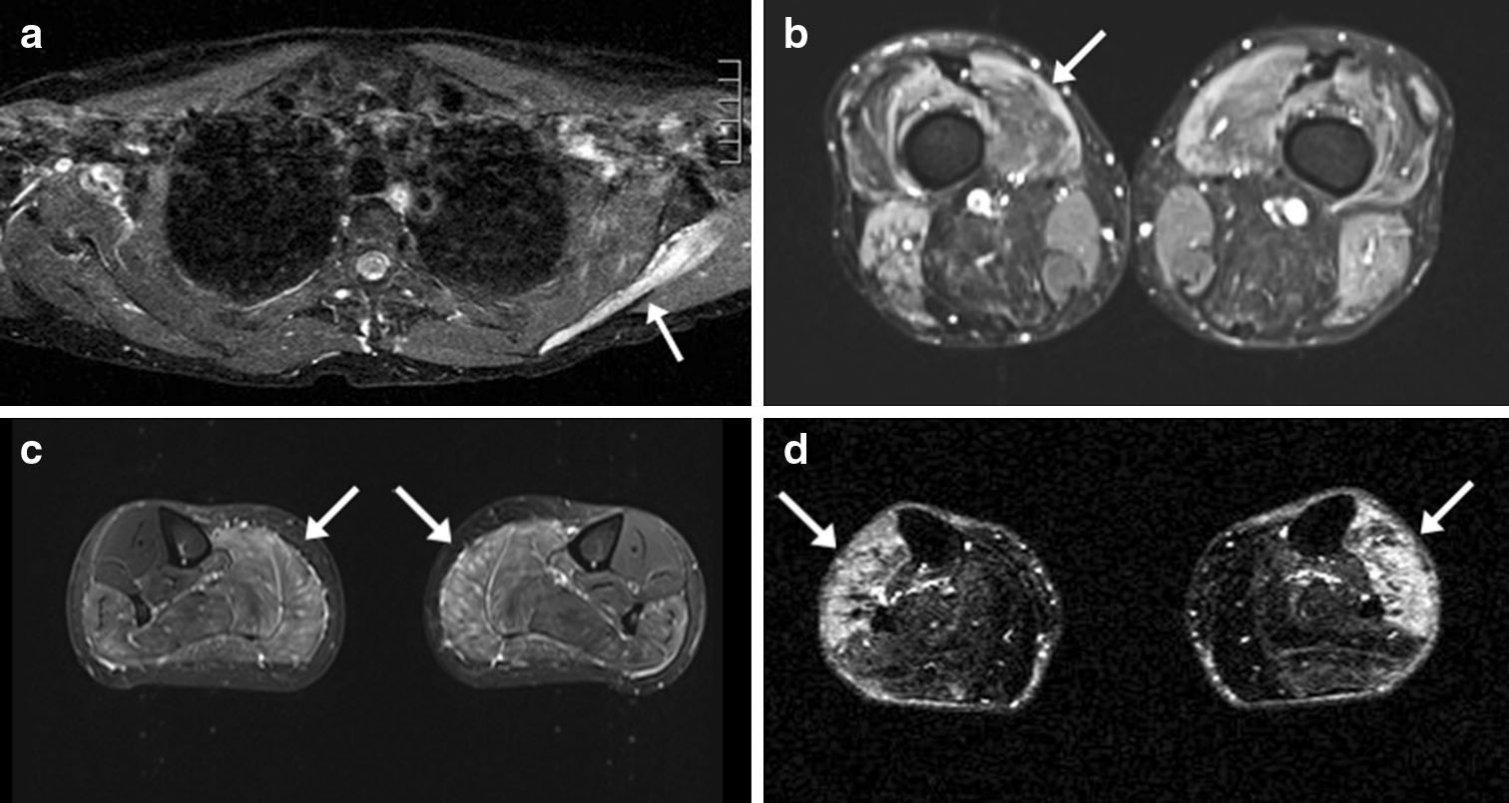
STIR positive images in NLSD-M and NLSD-I. STIR hyperintensities (*arrows*) in upper and lower limb muscles. STIR positive left infraspinatus in P1 (**a**), vastus medialis in P4 (**b**), posterior compartment of the legs in P12 (**c**) and anterior compartment of the legs in P6 (**d**)

In NLSD-M, upper limbs were less frequently involved than lower limbs. In the least affected patient (patient 10), only the lower limbs showed fatty replacement (Figs. 1, 3a). The most affected muscle in the neck and the scapular girdle was the infraspinatus, followed by supraspinatus, trapezius, deltoid and thoracic paraspinal muscles. Subscapularis, pectoralis minor and major, and sternocleidomastoid were the most spared muscles. Pectoralis major and sternocleidomastoid had a hypertrophic appearance in some cases. In all patients (4/10) with evaluable study of the proximal part of upper limbs, the anterior arm compartment showed a moderate to severe fatty replacement. By contrast triceps brachii was constantly spared. Two patients (P1 and P10) showed STIR hyperintensity in different muscle. Interestingly one of them (P1) had no fatty replacement in scapular girdle muscles, but showed a STIR positive image of infraspinatus suggesting that it could be the first affected muscle at the scapular girdle level (Figs. 3b, 4a). MRI images of the upper limbs were not available in the two NLSD-I subjects.

### Characteristics of fatty replacement

**Fig. 5:**
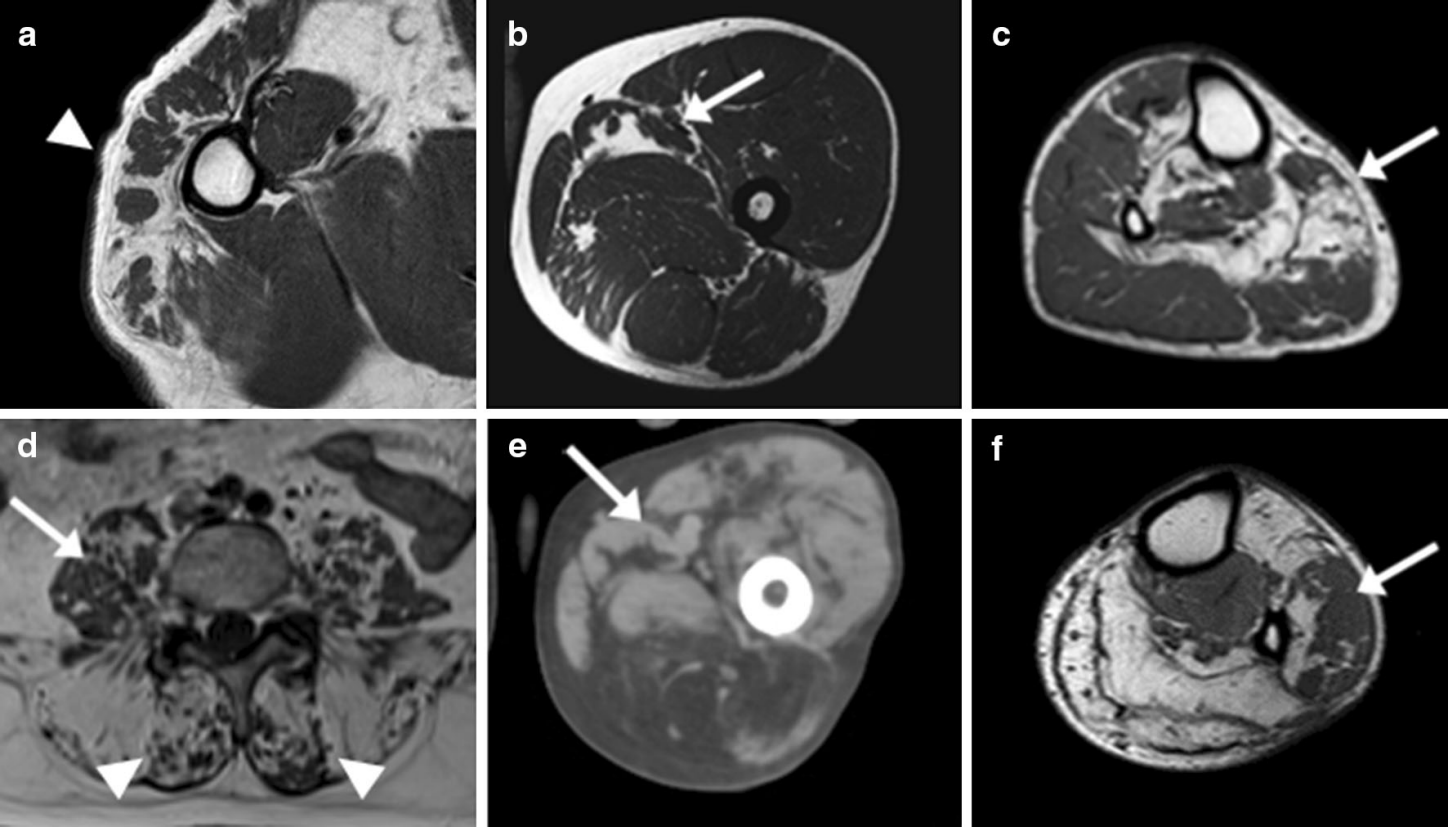
Patchy areas of fatty replacement in NLSD-M. T1-weighted images. **a**, **b**, **f** patient 2; **c** patient 5; **d** patient 4; **e** patient 7. Not homogenous muscle involvement in several muscles of different patients. Patchy areas of fatty replacement in bigger areas of not affected muscle (*arrows*) and muscle sparing areas in bigger fatty replaced areas (*arrowheads*)

Signal abnormalities were not homogeneous in all muscles. Patchy fat deposition (i.e., complete fatty replacement of discrete muscle areas close to areas of complete sparing) was present in at least one muscle in all the patients (Fig. 5), and it was more frequently observed in the lower limbs than in the upper limbs. The muscles more frequently showing this peculiar involvement were gluteus medius, adductor longus, and muscles of the antero-lateral compartment of the leg in the lower limbs. In the upper limbs, deltoid was the only one muscle where this inhomogeneous fatty replacement was observed.

### Symmetry

Muscle involvement was generally symmetric. Asymmetry of muscle involvement was observed in 4/10 NLSD-M patients, and specifically in 7 pairs of muscles in lower limbs and 1 pair in upper limbs. Asymmetry was detected more frequently in the legs (popliteus, both gastrocnemii, and tibialis posterior) than in the thighs. Minor asymmetry (1 point score of difference between the two sides) was more frequently observed. Globally asymmetry was more frequent in lower limbs than upper limbs.

## Discussion

We present a systematic study of muscle imaging in a large cohort of NLSD patients. Imaging data have been collected from patients of the Italian Network of NLSD, harboring different genetic mutations and different clinical disease severity. Previous data from single cases and smaller cohorts show results that are globally in agreement with the pattern of muscle involvement we have recognized in this work [4, 11, 12].

Even if the quality of imaging resolution by CT scan is lesser informative than MRI, the different degrees of muscle involvement was largely comparable between CT patients and MRI patients.

Despite clinical manifestations suggest a major clinical impairment of upper limbs in NLSD-M, muscle imaging demonstrated a more severe involvement of the lower limbs along the entire disease course (Figs. 1, 4). In the milder patient (P10), even if the upper limbs show no fatty replacement, an initial fatty replacement in lower limbs could be detected.

Medial gastrocnemius, soleus, gluteus minimus and semimembranosus are the most severely affected muscles in all patients. Moreover, leg (medial gastrocnemius and soleus) and pelvis (gluteus minimus) muscles were constantly more affected than thigh muscles (semimembranosus) suggesting that fatty replacement starts both at the pelvis and legs. Gluteus medius, biceps femoris (long head), adductor magnus and longus, gastrocnemius medialis and tibialis posterior appeared to be less severely affected. Notably, tibialis posterior is affected early in the disease course, contrary to the majority of myopathies, in particular LDMG and distal myopathies, in which tibialis posterior is frequently spared even in the late-end stages of the disease course [17, 25–29]. Muscles of anterior compartment of the leg are variably affected during the disease course while quadriceps and psoas become affected in the late-end stages of disease. Psoas sparing can help to recognize NLSD from other myopathies in the late-end course of the disease when specificity of the MRI pattern involvement disappears or the muscle biopsy may be not informative [17, 25–29]. Sparing of sartorius, gracilis and pectineus is constantly observed in all cases.

Once the upper limbs are involved, the first and constantly affected muscle is the infraspinatus. Supraspinatus, trapezius, deltoid, serratus and paraspinal muscles are also frequently affected, but a lesser degree. In the arms, the anterior compartment is markedly involved. By contrast sternocleidomastoid, pectoralis minor and major and triceps brachii are constantly spared in all cases and sometimes appear to be hypertrophied, differently from other reported myopathies [30].

Taken together, this combination of muscle involvement in lower and upper limbs composes a constant pattern and represents a signature of NLSD-M in all stages of the disease. In the clinical context, muscle MRI may help to recognize NLSD-M among different conditions affecting predominantly proximal upper limbs with neck extensor weakness (“man in the barrel” or “dropped head” syndromes) [31–34] or among different metabolic myopathies, notably those associated with lipidosis on the muscle biopsy [35]. Nevertheless, more muscle imaging data are needed from these other conditions to establish if the “MRI signature” in NLSD-M is specific when compared to other muscle lipidosis.

In comparison to other muscular dystrophies and in particular with LGMD, it is notable that in the late-end stages of disease, gracilis, sartorius and biceps femoris (short head) are frequently spared, but differently from NLSD-M, calf involvement appears constantly later than thigh involvement (BMD, sarcoglycanopathies and LGMD2I) [17, 29]. In dysferlinopathies (LGMD2B), even if posterior compartments of the thigh and calf are frequently involved, the quadriceps involvement, and particularly the vastus lateralis, appears early in the disease course, whereas in NLSD-M is constantly involved lesser than posterior compartment.

In distal myopathies, muscle replacement starts in the legs as in NLSD-M. Nevertheless, in Desminopathies semitendinosus is frequently early replaced and semimembranosus is spared until the end stage of the disease [25]. By contrast myotilinopathy and ZASP myopathy could show similar muscle involvement in the leg and thigh to NLSD-M even if lateral gastrocnemius is frequently spared [16]. Another myopathy with a similar pattern of myotilinopathy is LGMD1D, but peroneal compartment if frequently spared in this condition [36].

Another important finding highlighted by our work is the characteristic aspect of fatty replacement constantly observed in all NLSD-M patients, that had never been reported in previous studies concerning NLSD. Indeed, in several muscles, fatty replacement was not homogenous along the whole length of the muscles and in early stage of the disease “patchy” areas of total fatty replacement were close to areas of muscle sparing. Moreover, in the most affected patients, “islands” of muscle sparing were present in the middle of large areas of fatty replacement. This unusual pattern of fatty replacement could represent an additional “disease signature” of NLSD-M, and could reflect different pathophysiological mechanisms of disease compared to muscular dystrophies. Nevertheless more physiological studies are necessary to support this hypothesis.

In our NLSD-I patients, muscle involvement was very mild and only showed hyperintense STIR images in the calf muscles. Nevertheless, these data should be further assessed and confirmed in a larger cohort of patients, because the overall number of muscle MRI observations of NLSD-I patients is scanty [13].

In conclusion we describe the muscle imaging findings in a large cohort of NLSD patients. Our data provides evidence that muscle imaging can identify characteristic alterations for NLSD-M, such as a consistent pattern of muscle involvement associated to the presence of “spotted” areas of fatty replacement.

Larger cohorts are needed to assess if a distinct pattern of muscle involvement exists also for NLSD-I.

## Electronic supplementary material

Below is the link to the electronic supplementary material.

Supplementary material 1 (DOC 30 kb)
