# Supplementary material for: Muscle MRI in neutral lipid storage disease (NLSD)
Source: J Neurol. 2017 May 13;264(7):1334–42. doi: 10.1007/s00415-017-8498-8 (PMC5502068; doi:10.1007/s00415-017-8498-8)
Supplement: Supplementary file 1 — Supplementary material 1 (DOC 30 kb) [file 415_2017_8498_MOESM1_ESM.doc]

SUPPLEMENTARY MATHERIALS

List of examined muscles in lower limbs and scapular girdle:

**Lower limb muscles:**

1. Ileo Psoas
2. Gluteus Maximus
3. Gluteus Medius
4. luteus Minimus
5. Tensor Fasciae Latae
6. Sartorius
7. Gracilis
8. Rectus Femoris
9. Vastus Intermedius
10. Vastus Medialis
11. Vastus Lateralis
12. Biceps Femoris Caput Longus
13. Biceps Femoris Caput Breve
14. Semitendinosus
15. Semimembranosus
16. Adductor Magnus
17. Adductor Longus
18. Adductor Brevis
19. Pectineus
20. Obturatorius Internus
21. Obturatorius Externus
22. Popliteus
23. Gastrocnemius Medialis
24. Gastrocnemius Lateralis
25. Soleus
26. Tibialis Anterior
27. Extensor Digitorum Longus
28. Extensor Hallucis Longu
29. Peroneus Longus
30. Peroneus Brevis
31. Tibialis Posterior
32. Flexor Digitorum Longu
33. Flexor Hallucis Longus

**Scapular girdle muscles :**

1. Paraspinous (Cervical)
2. Levator Scapulae
3. Sternocleidomastoideus
4. Trapezius
5. Subscapularis
6. Supraspinatus
7. Infraspinatus
8. Serratus anterior
9. Romboidei
10. Deltoideus
11. Teres Major
12. Teres Minor
13. Latissimus Dorsi
14. Pectoralis Major
15. Pectoralis Minor
16. Paraspinous (Thoracic)
17. Triceps Brachii
18. Anterior arm muscles (Biceps Brachii, Coracobrachialis and Brachialis)
